# Prokineticin 1 induces a pro-inflammatory response in murine fetal membranes but does not induce preterm delivery

**DOI:** 10.1530/REP-13-0295

**Published:** 2013-12

**Authors:** Tamsin R M Lannagan, Martin R Wilson, Fiona Denison, Jane E Norman, Rob D Catalano, Henry N Jabbour

**Affiliations:** 1 MRC Human Reproductive Science UnitThe Queen's Medical Research Centre, University of Edinburgh47 Little France Crescent, Edinburgh, EH16 4TYUK; 2 MRC Centre for Reproductive HealthThe Queen's Medical Research Centre, University of Edinburgh47 Little France Crescent, Edinburgh, EH16 4TYUK

## Abstract

The mechanisms that regulate the induction of term or preterm delivery (PTD) are not fully understood. Infection is known to play a role in the induction of pro-inflammatory cascades in uteroplacental tissues associated with preterm pathological parturition. Similar but not identical cascades are evident in term labour. In the current study, we used a mouse model to evaluate the role of prokineticins in term and preterm parturition. Prokineticins are multi-functioning secreted proteins that signal through G-protein-coupled receptors to induce gene expression, including genes important in inflammatory responses. Expression of prokineticins (*Prok1* and *Prok2*) was quantified in murine uteroplacental tissues by QPCR in the days preceding labour (days 16–19). *Prok1* mRNA expression increased significantly on D18 in fetal membranes (compared with D16) but not in uterus or placenta. Intrauterine injection of PROK1 on D17 induced fetal membrane mRNA expression of the pro-inflammatory mediators *Il6*, *Il1b*, *Tnf*, *Cxcl2* and *Cxcl5*, which are not normally up-regulated until D19 of pregnancy. However, intrauterine injection of PROK1 did not result in PTD. As expected, injection of lipopolysaccharide (LPS) induced PTD, but this was not associated with changes in expression of *Prok1* or its receptor (*Prokr1*) in fetal membranes. These results suggest that although *Prok1* exhibits dynamic mRNA regulation in fetal membranes preceding labour and induces a pro-inflammatory response when injected into the uterus on D17, it is insufficient to induce PTD. Additionally, prokineticin up-regulation appears not to be part of the LPS-induced inflammatory response in mouse fetal membranes.

## Introduction

The factors that trigger the onset of parturition at term in humans (∼39 weeks) are not yet fully understood, although inflammation in uteroplacental tissues and increased priming of leukocyte activation in maternal peripheral blood are well documented (Peltier 2003, Haddad *et al*. 2006, Bollapragada *et al*. 2009, Challis *et al*. 2009, Yuan *et al*. 2009). The cervix and uteroplacental tissues (myometrium, placenta and fetal membranes) demonstrate elevated leukocyte numbers and cytokine production during parturition at term (Denison *et al*. 1998, Thomson *et al*. 1999, Young *et al*. 2002, Osman *et al*. 2003, 2006). Increased interleukin 1B (IL1B), IL6, IL8 and tumour necrosis factor (TNF) production by recruited and resident immune cells leads to induction of matrix metalloproteinases (MMPs) that facilitate cervical ripening and membrane rupture (Watari *et al*. 1999, Allport *et al*. 2001, Kelly 2002, Xu *et al*. 2002, Young *et al*. 2002, Timmons *et al*. 2010). In myometrium, IL1B, IL6 and TNF contribute to the onset and synchronisation of contractions by inducing calcium release and expression of cyclo-oxygenase (COX) enzymes, prostaglandin synthesis and expression of prostaglandin and oxytocin receptors (Friebe-Hoffmann *et al*. 2001, Tribe *et al*. 2003, Zaragoza *et al*. 2006, Fitzgibbon *et al*. 2009).

Preterm delivery (PTD) in humans is considered to occur before 37 weeks of gestation and is the largest cause of neonatal mortality and morbidity in the developed world (Norman *et al*. 2009, Beck *et al*. 2010). Although the aetiology is not fully understood, there are several factors that are associated with increased risk (Goldenberg *et al*. 2008) including inflammation/infection in the reproductive tract (Goldenberg *et al*. 2000). Inflammation in uteroplacental tissues can occur in response to bacterial invasion of the reproductive tract as part of the innate immune response (Romero *et al*. 2006) but also occurs in its absence (Tornblom *et al*. 2005). The likely sequence of events is that immune cells and tissues expressing toll-like receptors (TLRs) recognise pathogen-associated molecular patterns including lipopolysaccharide (LPS), a component of the cell wall of Gram-negative bacteria. LPS binding to TLR4 (along with CD14 and MD-2) leads to activation of the transcription factor NFKB, a major regulator of pro-inflammatory mediators (Akira *et al*. 2006, Doyle & O'Neill 2006), resulting in the premature expression of cytokines and chemokines in gestational tissues. This process culminates in early onset of cervical ripening, uterine contractions and PTD. In addition, premature inflammation in uteroplacental tissues is implicated in fetal inflammatory injury, an adverse neonatal outcome (Goepfert *et al*. 2004). Current therapies for preterm labour attempt to delay the onset of parturition; however, additionally targeting the inflammatory response could not only delay parturition but also reduce fetal inflammatory injury.

Fetal membranes in humans comprise amnion and chorion and an extracellular matrix. At term parturition the fetal membranes overlying the cervix rupture; evidence suggests that this is due to reduced collagen levels and increased apoptosis. However, a role for fetal membrane activation in PTD has also been described as clinical and histological chorioamnionitis (inflammation of the amnion and chorion) can lead to preterm premature rupture of membranes (pPROM) (reviewed by Menon & Fortunato (2007) and Gomez-Lopez *et al*. (2010)).

PTD can be artificially induced by treating pregnant mice with LPS (via the i.p., intrauterine, intra-amniotic or vaginal routes), allowing the effect of preterm labour (and potential therapeutic targets) on neonatal morbidity and mortality to be investigated (Elovitz *et al*. 2003, Pirianov *et al*. 2009, Burd *et al*. 2010, Gonzalez *et al*. 2011).

*In silico* data (Catalano *et al*. 2010) suggest that another target of NFKB transcriptional regulation in response to LPS could be the multi-functional secreted proteins prokineticin 1 (PROK1) and PROK2 and their G-protein-coupled receptors (GPCRs: PROKR1 and PROKR2). Prokineticins can regulate a wide variety of biological processes including angiogenesis, haematopoiesis, nociception, circadian rhythms, smooth muscle contractility and inflammatory responses (LeCouter *et al*. 2001, Li *et al*. 2001, Negri *et al*. 2002, Cheng *et al*. 2005, Dorsch *et al*. 2005, Maldonado-Perez *et al*. 2007, Monnier & Samson 2008, Ngan & Tam 2008). In relation to pregnancy and parturition, it is also known that prokineticins play a role in establishing early pregnancy (Evans *et al*. 2008, 2009, Waddell *et al*. 2011) and in pathological conditions such as pre-eclamspia and ectopic pregnancy (Hoffmann *et al*. 2006, 2009, Shaw *et al*. 2010). In addition, we have recently demonstrated that prokineticins are up-regulated at term in uteroplacental tissues and that *ex vivo* treatment with PROK1 induces a cascade of pro-inflammatory pathways in human placenta and myometrial tissue collected from pregnant women at term (Denison *et al*. 2008, Gorowiec *et al*. 2011). These data taken together demonstrate the potential role for prokineticins in regulating inflammatory pathways associated with term parturition and therefore their possible involvement in preterm labour.

This study was designed to characterise the expression of prokineticins in mouse uteroplacental tissues in the days preceding labour and to investigate the role of prokineticins in term and preterm parturition using a mouse model of infection-induced PTD .

## Materials and methods

### Mouse model of PTL

CD1 outbred, timed pregnant mice were purchased from Charles River (Margate, Kent, UK) and allowed at least 6 days of acclimatisation prior to surgery. Pregnant females or virgin female and proven males were purchased pregnant and timed-mated within the University of Edinburgh facility. Surgery was performed on day 17 of pregnancy (D17; day plug found is D1 of pregnancy). Dams were anaesthetised with isoflurane and both uterine horns exposed following laparotomy. The number of viable fetuses in each horn was recorded and the horn containing the most fetuses was selected for intrauterine injection. Using a 33-gauge Hamilton syringe, pregnant mice were injected between the two most anterior fetuses with either a 25 μl volume of sterile saline (Sigma); recombinant mouse PROK1 (R&D Systems, Abingdon, Oxford, UK) diluted to 350 nM in saline (dose based on highest concentration shown to induce cell proliferation in bovine endothelial cells *in vitro*, ED_50_ of 4 μg/ml, determined by R&D Systems, and nearly ten times the dose that induces cytokine expression in human gestational tissues (Maldonado-Perez *et al*. 2009); or 20 μg LPS (Sigma) in saline (see Table 1 for summary of treatments and sample sizes). The body wall was closed using a continuous suture and the skin closed using single stitches. Dams received Vetergesic analgesia (Reckitt Benckiser Healthcare, Kingston-upon-Thames, UK) and were allowed to recover in a warm environment prior to single housing and monitoring for delivery (infra-red cameras and digital video recorder purchased from RF Concepts (Dundonald, UK)). The time to delivery was the number of hours recorded from injection to delivery of the first pup. To record the time taken for natural delivery (in the absence of surgery), the average time of saline injection was calculated and used as a theoretical time point from which to calculate the time to delivery. Pup survival was calculated as the number of live pups found in the cage (or dam) at the time of culling as a proportion of the total number of viable fetuses recorded at injection. Animals were housed under standard conditions and had access to food and water *ad libitum*. All animal care and experimental protocols were approved by the animal ethics committee of the University of Edinburgh and the Home Office of the UK government.

### Amniotic fluid collection, tissue harvesting and processing

Tissue was collected from timed-mated CD1 mice on D16–19 of pregnancy (parturition occurred on D20). Dams were culled by cervical dislocation and uterus, placenta and fetal membranes separated, and collected into RNAlater (Ambion (Life Technologies Ltd, Paisley, UK)) for RNA extraction or fixed in 4% neutral buffered formalin and wax embedded for immunohistochemistry (see Table 2 for summary of treatments and sample sizes). Similar collection protocols were applied 2 or 6 h after intrauterine injection of saline, LPS or PROK1 (as described earlier for the mouse model of PTD). In addition, for the 6-h time point, amniotic fluid was collected and pooled from four to six fetuses (i.e. one sample per dam).

### TaqMan quantitative RT-PCR

Total RNA was extracted from tissue using QIAzol lysis reagent, phase lock tubes and the RNeasy mini kit with on-column DNase digestion from Qiagen, according to the manufacturer's guidelines. RNA concentration was quantified using a Nanodrop (Thermo Scientific, Hemel Hempstead, UK) and diluted to 100 ng/μl in RNase-free water (Invitrogen) before RT using the Superscript VILO cDNA synthesis kit (Invitrogen). PCRs were carried out using Applied Biosystems 7500 and 7900 Fast instruments. Primer and FAM (6-carboxyfluorescein)-labelled probe sequences were designed to span an intron (where possible) and purchased from Sigma (Table 3) or TaqMan Gene Expression Assays were purchased from Applied Biosystems (Table 4). Expression was normalised for RNA loading using actin (*Actb*) primers designed to span an intron and JOE-labelled probe (Sigma; Table 3). Relative expression in each sample was calculated against the level of expression detected in a tissue known to express the target gene (Tables 3 and 4). To compare the changes in pro-inflammatory mediator expression in response to intrauterine injection of LPS or PROK1 with those occurring endogenously at term, fold changes were calculated by dividing the mean relative expression in the treated group by the mean relative expression in the saline group, and by dividing mean relative expression on D19 with that on D17 (the day on which surgery was carried out).

### Immunohistochemistry

Paraffin-embedded tissue was processed for immunohistochemistry as described in Catalano *et al*. (2011) with either rabbit anti-human PROK1 at 1:750 dilution (Phoenix Pharmaceuticals (Belmont, CA, USA), H-023-59) or rabbit anti-human PROKR1 at 1:750 dilution (Caltag Medsystems, Buckingham, UK; LS-A6684). Negative controls were incubated without primary antibody. Sections were imaged using a Provis AX70 microscope (Olympus, Southend-on-Sea, UK) and photographed using an AxioCam HRc with AxioVision Release 4.8 (Carl Zeiss Ltd., Berlin, Germany).

### Cytokine assays

Amniotic fluid collected from saline-, LPS- or PROK1-treated dams was quantified by ELISA Quantikine Kits (R&D Systems) for mouse IL1B (MLB00B), TNF (MTA00) and CXCL2 (MM200) as per kit instructions.

### Statistical analysis

Data that passed the normality test (Kolmogorov–Smirnov) were subjected to statistical analysis using a one-way ANOVA with Tukey's multiple comparison post-test or a two-tailed unpaired *t*-test. Nonparametric data were subjected to Kruskal–Wallis test with Dunn's post-test or a two-tailed Mann–Whitney *U* test. Percentages were arcsine transformed prior to analysis as they cannot be normally distributed (no values <0 and nothing >100). Where more than one fetus was collected per dam the data was averaged per dam. All analysis was carried out using GraphPad Prism and GraphPad Software, Inc. (La Jolla, CA, USA). Significance was defined as *P*<0.05.

## Results

### Expression of prokineticins in murine uteroplacental tissues across the days preceding parturition

**Figure 1:**
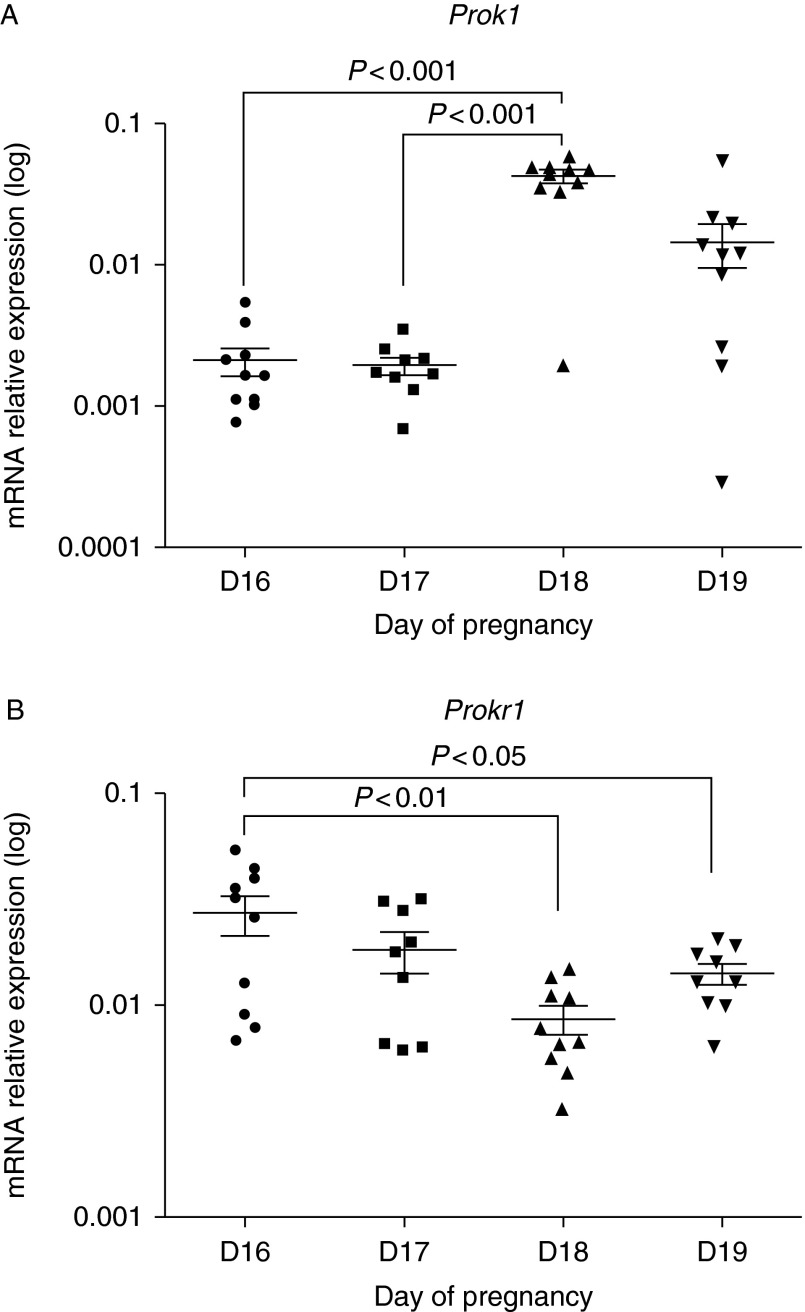
*Prok1* mRNA expression peaks on D18 of pregnancy in murine fetal membranes. QPCR expression analysis of *Prok1* (A) and *Prokr1* (B) in murine fetal membranes reveals dynamic regulation preceding labour (D16–19). The graphs show individual values for each sample (one membrane analysed per mouse), mean expression levels are in arbitrary units normalised to *Actb* mRNA, error bars represent±s.e.m. (ANOVA). D16 (*n*=10), D17 (*n*=9), D18 (*n*=10) and D19 (*n*=10). Note: logarithmic scale.

Expression of *Prok1* and *Prokr1* mRNA was examined daily in fetal membranes from D16 to 19 of pregnancy (animals delivered on D20 of pregnancy; one fetal membrane from a minimum of nine to ten dams). On D18, expression of *Prok1* mRNA was significantly greater when compared with expression on D16 and 17 (*P*<0.001; Fig. 1A) and *Prokr1* expression was significantly lower on D18 and 19 when compared with D16 (*P*<0.01 and *P*<0.05 respectively; Fig. 1B).

**Figure 2:**
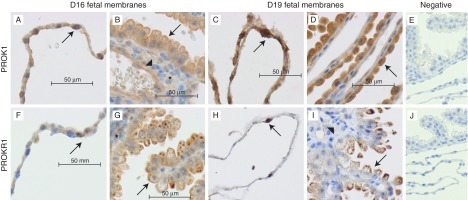
PROK1 and PROKR1 are expressed at the protein level by murine fetal membranes preceding labour. Immunohistochemical localisation of PROK1 (A, B, C and D) and PROKR1 (F, G, H and I) in D16 (A, B, F and G) and D19 (C, D, H and I) murine fetal membranes revealed that PROK1 is localised in the epithelium of amnion and yolk sac (see arrows in (A, B, C and D)) and in mesenchyme (asterisk) and endothelium (arrowhead) of the yolk sac (B). PROKR1 is expressed in the epithelium of amnion and yolk sac (see arrows in (F, G, H and I)) and in mesenchyme (asterisk in (G)) and endothelium of yolk sac (arrowhead in (I)). (E and J) Negative controls for PROK1 and PROKR1 respectively.

Protein expression of PROK1 and PROKR1 in fetal membranes was investigated by immunohistochemistry between D16 and 19. No change in localisation was observed during this period, although PROK1 expression was stronger in D19 fetal membranes than those in D16 (Fig. 2, D17–18, not shown). PROK1 and PROKR1 were localised in the amnion epithelium (Fig. 2A, C, F and H). In the yolk sac (considered to be equivalent to the human chorion), PROK1 and PROKR1 were localised to the epithelium, mesoderm and endothelial cells (Fig. 2B, D, G and H).

Expression analysis of *Prok1* in the uterus and placenta revealed no significant change across D16–19 (one tissue sample from a minimum of four to five dams; Supplementary Figure 1A and C, see section on supplementary data given at the end of this article). *Prokr1* expression decreased towards the end of gestation in the uterus (D19 vs D16 and 17, *P*<0.05; Supplementary Figure 1B) and placenta (D19 vs 17, *P*<0.05; Supplementary Figure 1D).

Expression of *Prok2* and *Prokr2* was also examined in the same uteroplacental tissue samples from D16 to 19. Fetal membrane *Prok2* expression increased by a small but significant amount on D18 when compared with expression on D16 (*P*<0.05; Supplementary Figure 2A, see section on supplementary data given at the end of this article). No changes were detected for *Prokr2* in the fetal membranes (Supplementary Figure 2B). No significant changes were detected for either *Prok2* or *Prokr2* in the uterus and placenta (Supplementary Figure 2C, D, E and F).

### Intrauterine injection of LPS or PROK1 enhances expression of pro-inflammatory mediators in fetal membranes

**Figure 3:**
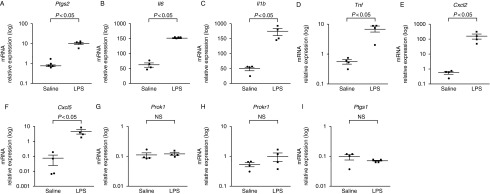
Intrauterine injection of LPS induces a pro-inflammatory response in murine fetal membranes but does not regulate *Prok1* or *Prokr1*. QPCR mRNA expression analysis of (A) *Ptgs2*, (B) *Il6*, (C) *Il1b*, (D) *Tnf*, (E) *Cxcl2*, (F) *Cxcl5*, (G) *Prok1*, (H) *Prokr1* and (I) *Ptgs1* in murine fetal membranes 6 h after intrauterine injection of LPS reveals up-regulation of pro-inflammatory mediators but not of *Prok1*, *Prokr1* or *Ptgs1* (negative control). The graphs show mean values for each dam (three membranes analysed per dam), mean expression levels are in arbitrary units normalised to *Actb* mRNA, error bars represent±s.e.m. (*t*-test). Saline (*n*=4 dams) and LPS (*n*=4 dams). NS, non-significant. Note: logarithmic scale.

**Figure 4:**
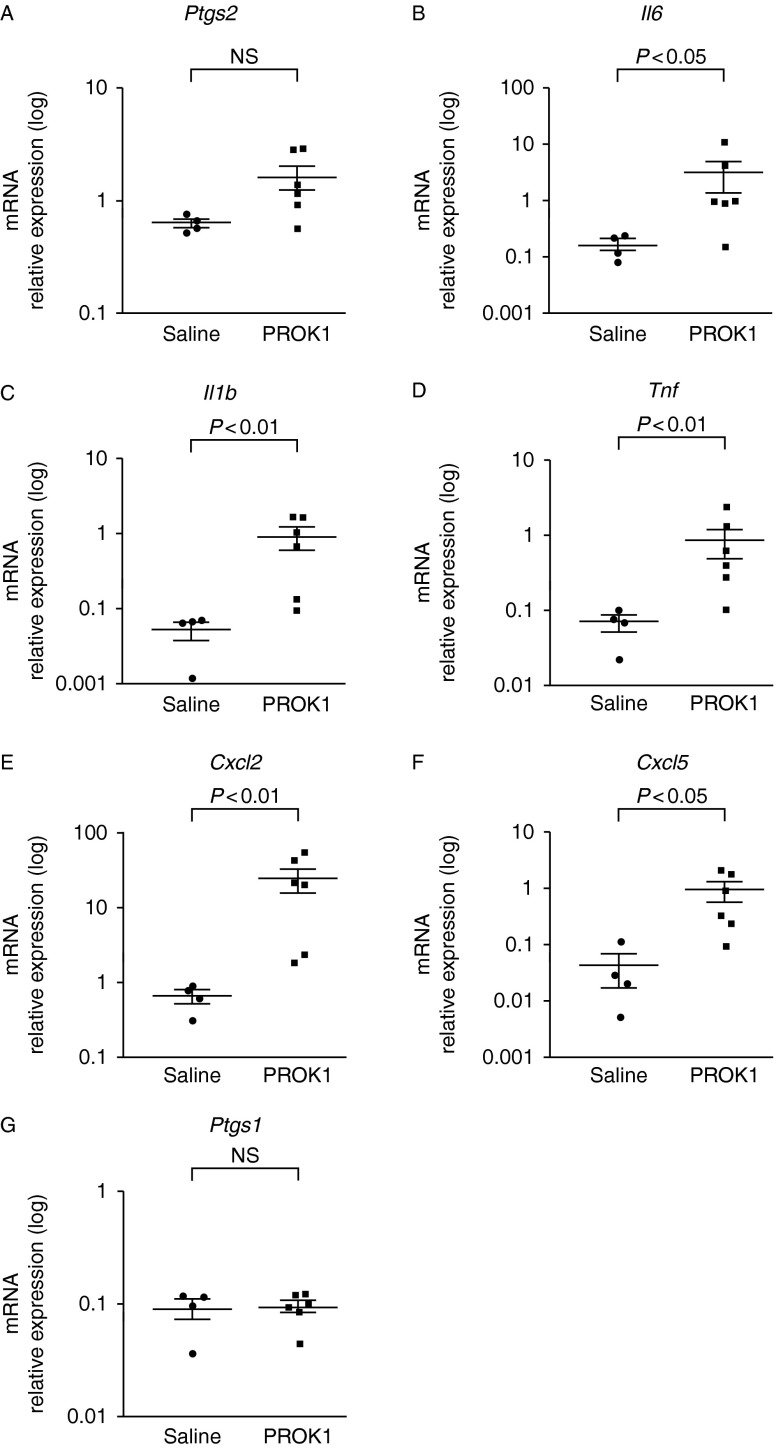
Intrauterine injection of PROK1 induces a pro-inflammatory response in murine fetal membranes. QPCR expression analysis of pro-inflammatory mediators (B) *Il6*, (C) *Il1b*, (D) *Tnf*, (E) *Cxcl2* and (F) *Cxcl5* in murine membranes 6 h after intrauterine injection of PROK1 reveals their up-regulation but not of (A) *Ptgs2* or (G) *Ptgs1* (negative control). Each data point represents the mean response for each dam (three membranes per dam) and the graphs show the mean response per treatment group. Mean expression levels are in arbitrary units normalised to *Actb* mRNA, error bars represent±s.e.m. (*t*-test). Saline (*n*=4 dams) and PROK1 (*n*=6 dams). NS, non-significant. Note: logarithmic scale.

LPS treatment is well documented to induce pro-inflammatory responses in many tissues. In this study, we investigated the pro-inflammatory response of fetal membranes to LPS and PROK1 *in vivo* using a model of infection-induced PTD. For this, fetal membranes were collected 6 h after intrauterine injection with saline (control; average of three fetal membranes collected from each of four dams), LPS (average of three fetal membranes collected from each of four dams) or PROK1 (average of three fetal membranes collected from each of six dams). Compared with the saline treatment, relative expression of prostaglandin synthase 2 (*Ptgs2*, also referred to as COX2), *Il6*, *Il1b*, *Tnf*, chemokine (CXC motif) ligand 2 (*Cxcl2*) and *Cxcl5* mRNAs were found to be significantly increased in response to LPS treatment (Fig. 3). By contrast, expression of *Ptgs1* (a gene chosen as a negative control) did not change in response to treatment with LPS. Similarly, *Prok1* and *Prokr1* expression levels were not regulated by LPS treatment at the 6-h time point (Fig. 3) or earlier at 2 h (data not shown). Intrauterine administration of PROK1 significantly increased expression of *Il6*, *Il1b*, *Tnf*, *Cxcl2* and *Cxcl5* but not *Ptgs2* or *Ptgs1* by 6 h after treatment (Fig. 4).

We subsequently investigated the temporal expression of *Ptgs2*, *Il6*, *Il1b*, *Tnf*, *Cxcl2* and *Cxcl5* between D16 and 19 of pregnancy and all were shown to significantly increase on D19 in fetal membranes (Supplementary Figure 3, see section on supplementary data given at the end of this article). Using relative expression values, we compared the endogenous changes occurring in fetal membranes at the onset of parturition with those occurring in response to PROK1 or LPS (Table 5). Endogenous levels on D19 were compared with levels on D17. Apart from *Tnf*, overall expression of pro-inflammatory genes in response to intrauterine injection of 350 nM PROK1 was comparable (less than twofold greater) or lower than seen endogenously on D19 of pregnancy. Intrauterine injection of 20 μg LPS resulted in a greater fold increase in expression of pro-inflammatory genes compared with PROK1 and those measured endogenously on D19 (apart from *Cxcl5*). Interestingly, it was the chemokines *Cxcl2* and *Cxcl5* that showed the most striking differential expression in response to either term parturition or LPS treatment.

### Intrauterine injection of LPS but not PROK1 regulates production of pro-inflammatory mediators in amniotic fluid

**Figure 5:**
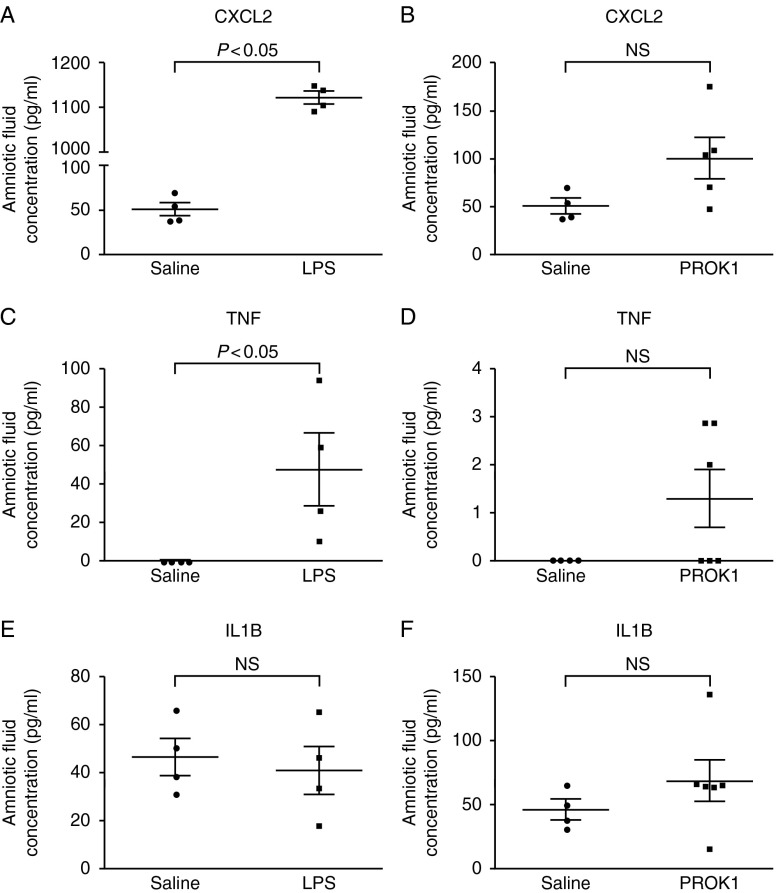
Intrauterine injection of LPS but not PROK1 increases the levels of secreted cytokines in murine amniotic fluid. Protein quantification of IL1B, TNF and CXCL2 by ELISA in amniotic fluid 6 h after intrauterine injection of LPS (A, C and E) or PROK1 (B, D and F) reveals significant up-regulation of TNF and CXCL2 in response to LPS but not PROK1 and no increase in IL1B to either treatment. Each data point represents the mean response for each dam (amniotic fluid from four to six sacs per dam) and the graphs show the mean response per treatment group. Mean expression levels (pg/ml), error bars represent±s.e.m. (*t*-test). NS, non-significant.

To compare the pro-inflammatory stimuli (LPS and PROK1), levels of secreted IL1B, TNF and CXCL2 were measured by ELISA in amniotic fluid collected 6 h after intrauterine injection (amniotic fluid was pooled from the amniotic sacs of four to six fetuses). Unexpectedly, a different pattern of results was obtained for each protein. IL1B was not regulated by either treatment, although PROK1 did demonstrate a trend for higher levels than LPS (saline, 46.60±7.72 pg/ml; PROK1, 69.12±15.85 pg/ml and LPS, 41.03±9.96 pg/ml; Fig. 5A). All samples from saline-treated animals contained undetectable levels of TNF, as did half of the PROK1-treated samples (saline, 0±0 pg/ml and PROK1, 1.30±0.60 pg/ml). By contrast, all LPS-treated samples contained detectable TNF (47.95±18.67 pg/ml) and the mean was significantly higher than the saline control (*P*<0.05; Fig. 5B). Measurement of CXCL2 revealed a similar pattern to TNF, with PROK1-treated samples demonstrating a trend for greater concentration than saline-treated samples (although not significant; saline, 50.57±7.60 pg/ml and PROK1, 101.00±21.33 pg/ml) while the LPS-treated samples contained a significantly higher concentration (1121.08±13.63 pg/ml; *P*<0.01).

### Intrauterine injection of PROK1 does not induce PTD

**Figure 6:**
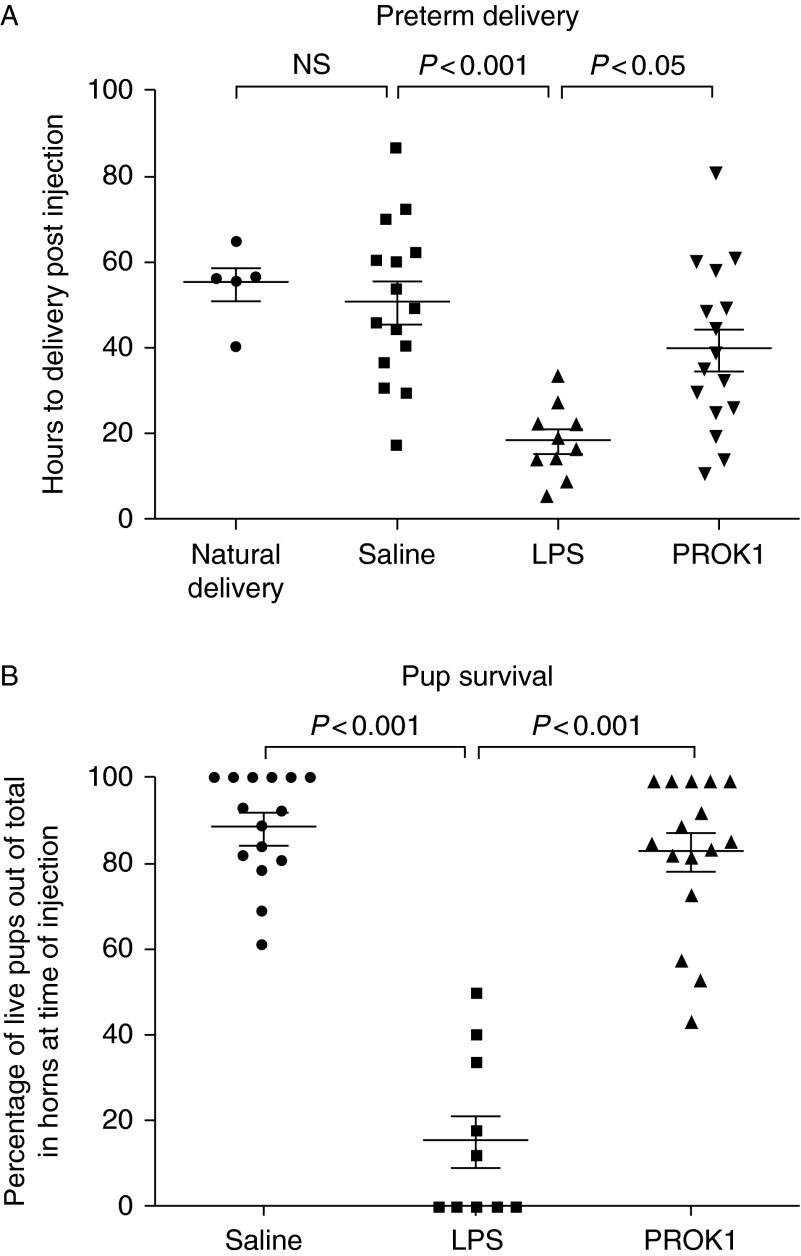
Intrauterine injection of PROK1 does not induce PTD. Time to delivery of first pup (A) and percentage pup survival per dam (B) following intrauterine injection of saline, LPS (20 μg) or PROK1 (350 nM) were compared. The number of hours to natural delivery (nonsurgical) was not significantly different from saline-treated dams but LPS treatment significantly reduced the time to delivery. Error bars represent±s.e.m. (ANOVA). Natural delivery (*n*=5 dams), saline (*n*=15 dams), LPS (*n*=10 dams) and PROK1 (*n*=16 dams). NS, non-significant.

In order to investigate the effect of PROK1 on PTD, three groups of mice received intrauterine injection of saline, LPS or PROK1 on D17 of pregnancy (Fig. 6A). The time to delivery for mice treated with saline was 50.50±4.79 h (*n*=15). The time to delivery in dams treated with PROK1 (39.19±4.87 h; *n*=16) was not significantly different from the time to delivery in dams treated with saline. However, treatment with LPS significantly reduced the time to delivery (17.90±2.65 h; *n*=10) compared with treatment with saline (*P*<0.001) or PROK1 (*P*<0.05). To assess whether or not surgery itself affected time to delivery, the time taken for five dams to naturally deliver was recorded (54.24±4.00 h; not significantly different from the saline-treated group).

Another measure of the effect of infection-induced PTD is to record pup survival (Fig. 6B). The average percentage of pups surviving per dam (calculated from the number of live pups in the cage or in the dam at time of culling) was significantly reduced in mice treated with LPS (15.33±6.08%) compared with treatment with saline or PROK1 (88.57±3.22% and 82.87±4.46% respectively; *P*<0.001).

## Discussion

In humans, term parturition and PTD are considered pro-inflammatory events (Bowen *et al*. 2002, Challis *et al*. 2009, Catalano *et al*. 2010, Golightly *et al*. 2011). *PROK1* mRNA expression is increased in human placenta and myometrium at term, and *ex vivo* treatment of these tissues with PROK1 induces a pro-inflammatory response (Denison *et al*. 2008, Gorowiec *et al*. 2011). *Ex vivo* treatment of term human myometrium with LPS up-regulates expression of *PROK1* and *PROKR1* (Gorowiec *et al*. 2011). In addition, PROK1 is a potent stimulator of smooth muscle contractions in the gut (Li *et al*. 2001, Wade *et al*. 2010). We hypothesised that PROK1 could be a potential mediator of pro-inflammatory pathways in human labour at term and may directly induce smooth muscle (myometrial) contractions. Thus, PROK1 induction may be part of the infection-induced pro-inflammatory response that contributes to PTD and antagonism of PROK1 could be a therapeutic target in the treatment of PTD.

We used the mouse as a model to investigate the role of prokineticins in parturition and PTD. Previously in the mouse, analysis of prokineticin expression in uteroplacental tissues has been restricted to the placenta (Hoffmann *et al*. 2007). Protein and mRNA expressions of *Prok1* and *Prokr1* were examined on 9.5, 10.5, 14.5 and 17.5 days post-coitus (dpc) and expression of both was significantly down-regulated after 10.5dpc, indicating a role in early placentation (Hoffmann *et al*. 2007). Therefore, we carried out the first daily examination of prokineticin expression in uteroplacental tissues preceding parturition in the mouse. Our data demonstrate that there is a dramatic up-regulation of *Prok1* mRNA in fetal membranes on D18 of pregnancy, but unlike human tissue at term, there was no increase in uterine or placental *Prok1*. We confirmed localisation of the ligand PROK1 and the receptor PROKR1 in mouse fetal membranes by immunohistochemistry; thus, endogenous PROK1–PROKR1 signalling could occur in an autocrine and paracrine fashion. In addition, the stronger PROK1 stain observed on D19 indicates that the increase in *Prok1* at the mRNA level is translated to increased protein expression. The up-regulation of *Prok1* mRNA and protein suggests an early role for PROK1 signalling in mouse parturition.

Parturition can be prematurely induced in mice by intrauterine injection of a mimetic of infection such as LPS (Pirianov *et al*. 2009). Intrauterine injection mimics the major route of pathogen transmission in humans, ascending from the vagina and cervix to reach uteroplacental tissues where a local immune response is induced leading to immune cell recruitment, synthesis of prostaglandins, MMPs and ultimately PTD. Fetal membranes are an attractive uteroplacental tissue to target in the therapeutic treatment of PTD as infection or inflammation in fetal membranes will result in bi-directional release of pro-inflammatory mediators into the amniotic fluid (and fetus, potentially causing fetal inflammatory injury) or towards placenta and uterus contributing to the initiation of parturition (Myatt & Sun 2010, Thiex *et al*. 2010).

In this study, we demonstrated that intrauterine treatment with PROK1 or LPS induces significant up-regulation of pro-inflammatory mediators in fetal membranes (*Il6*, *Il1b*, *Tnf*, *Cxcl2* and *Cxcl5*). We also demonstrated that these pro-inflammatory genes are significantly up-regulated in fetal membranes on D19 of pregnancy (the day before parturition). Indeed, *in vivo* PROK1-mediated induction of *Ptgs2*, *Il6* and *Il1b* cytokines by 6 h after treatment was comparable to that seen endogenously on D19 of pregnancy. These pro-inflammatory mediators are well documented to have important roles in term labour and PTD; for example, PTGS2 converts arachidonic acid into prostaglandins, which stimulate uterine smooth muscle contractions necessary for parturition (Challis *et al*. 1997), and inhibition of *Ptgs2* significantly reduces LPS-induced PTD (Gross *et al*. 2000). Mice null for *Il6* exhibit delayed labour and a reduced sensitivity to LPS-induced PTD (Robertson *et al*. 2010). *Il1b* mRNA levels increase in mouse uteroplacental tissues at term (Sato *et al*. 2001), and mice null for *Il1b* exhibit a reduced cytokine response to LPS (Reznikov *et al*. 1999). These data suggest that one possible role for endogenous PROK1 in fetal membranes could be to induce expression of other pro-inflammatory mediators leading to the induction of parturition at term. By contrast, our *in vivo* studies suggest that the up-regulation of pro-inflammatory mediators induced by exogenous PROK1 is insufficient to induce PTD. This may be because PTD requires greater production of pro-inflammatory mediators (for example at levels induced by LPS) in order to overcome any endogenous ‘brake’ on PTD.

This study identified that in response to LPS or PROK1 treatment, fetal membrane *Cxcl2* expression was more highly up-regulated than *Cxcl5*. Both are potent neutrophil CXC chemokines known to increase in response to LPS/infection in rodents and humans (Keelan *et al*. 2004, Harju *et al*. 2005). By contrast, at term parturition (D19 of pregnancy), the increase in *Cxcl5* expression was greater than that in *Cxcl2*. *CXCL5* is expressed by epithelium in response to IL1B and TNF in humans (Keelan *et al*. 2004). Therefore, the increase in murine *Cxcl5* in fetal membranes on D19 of pregnancy seems likely to reflect increased expression by fetal membrane epithelia; by contrast, *Cxcl2* is produced by macrophages. Intriguingly, neutrophil depletion does not affect time to delivery (Timmons & Mahendroo 2006) whereas macrophage depletion does prevent LPS-induced PTD (Gonzalez *et al*. 2011). Taken together, these data suggest that macrophage infiltration and elevated *Cxcl2* expression correlate with PTD, whereas elevated *Cxcl5* expression correlates with term parturition. Given that both CXCL2 and CXCL5 signal through the GPCR CXCR2, further research is warranted to investigate these chemokines as it may provide insight into the difference between the regulation of term parturition and PTD.

We also identified important differences between mouse and human prokineticin involvement in term labour and infection-induced PTD. Human myometrium and placenta (but not fetal membranes) exhibit a significant increase in *PROK1* expression at labour (Gorowiec *et al*. 2011). In addition, LPS increases expression of *PROK1* and *PROKR1* in human uteroplacental tissues (Gorowiec *et al*. 2011). By contrast, here we found that only mouse fetal membranes showed a significant elevation of *Prok1* preceding labour, and *in vivo* administration of LPS did not regulate murine expression of *Prok1* or *Prokr1* within 6 h. These data suggest that PROK1 and PROKR1 in mouse fetal membranes are not among the early complement of LPS-induced cytokines. This finding corroborates with our result that although PROK1 itself is pro-inflammatory, intrauterine injection of PROK1 is not sufficient to induce PTL. This result may be due to the lack of significant up-regulation of the inflammatory markers at the protein level as demonstrated by ELISA for CXCL2, TNF and IL1B in amniotic fluid 6 h after treatment. It is possible that a later time point may have yielded a different result as there is a trend for increased protein expression in response to PROK1 and it was found that LPS did not significantly up-regulate IL1B in amniotic fluid by 6 h.

Although the interval to birth was shorter after PROK1, this was not statistically significant. A *post hoc* power calculation shows that we would need 21 mice in each of the natural delivery groups and the PROK1 group to have 90% power to show a difference (at the 5% significance level) in the interval to delivery in the two groups. This assumes equal numbers in each groups and that the mean and s.d. of the time to delivery remain the same in each group. Hence, our study is underpowered to determine whether the relatively subtle effect of PROK1 on shortening time to delivery by 28% is statistically significant. The effect of LPS was profound, shortening the interval to delivery by 74%. Our study had 89% power to detect a shortening effect of PROK1 of time to delivery by 37%. We can be confident that any effect of PROK1 on inducing PTD (if it exists) is less than half as powerful as LPS in shortening the interval to delivery (in the doses given). In contrast to the possible type II error for a subtle effect of PROK1 on inducing PTD, there was no difference in pup survival between the PROK1 and the saline groups; thus, even if there is a modest effect of PROK1 on PTD, it does not compromise offspring survival.

At term, both humans and mice demonstrate elevated maternal inflammatory responsiveness while maintaining a relatively quiescent *in utero* environment (Orsi *et al*. 2007, Yuan *et al*. 2009). It has been hypothesised that this allows rapid induction of labour by priming the maternal inflammatory response to a trigger of parturition while protecting the fetus from fetal inflammatory injury that could be caused by premature exposure to inflammation. We have shown that classic markers of parturition increase significantly in fetal membranes on the day prior to labour (D19) while *Prok1* increases on D18; therefore, the ability of PROK1 to induce expression of these pro-inflammatory markers suggests that the endogenous increase in *Prok1* expression in fetal membranes on D18 may lead to induction of pro-inflammatory mediator expression and labour. Why we were unable to induce this with exogenous treatment on D17 remains unclear, although it is possible that a higher dose of PROK1 would have been more effective.

It has been demonstrated in fetal sheep lung that inflammation and immune cell recruitment in response to LPS treatment promotes lung maturation (Kallapur *et al*. 2005). This is an important factor in the survival and morbidity of the neonate (Jobe & Ikegami 2001) and may even be a trigger for parturition produced by the fetus (Condon *et al*. 2004, Harju *et al*. 2005). Thus, the endogenous increase in *Prok1* and its relatively mild pro-inflammatory properties (compared with LPS) may promote fetal maturation and potentially the induction of term labour. This hypothesis is consistent with our finding that PROK1 treatment, although pro-inflammatory, did not reduce pup survival. We can also speculate that as time to delivery was unaffected in PROK1-treated dams, the endogenous pro-inflammatory role of PROK1 is not to induce parturition through induction of a strong inflammatory response but to promote fetal maturation. This hypothesis may be supported by our finding that *Prok1* expression peaks on D18, and not on D19 like the other pro-inflammatory mediators analysed, suggesting that in the mouse, transient endogenous expression occurs rather than a prolonged amplification of pro-inflammatory mediator expression driven by PROK1 as hypothesised to occur in humans (Catalano *et al*. 2010, Gorowiec *et al*. 2011).

In conclusion, we have shown that PROK1 has a pro-inflammatory effect on fetal membranes but intrauterine injection is insufficient to induce PTD. However, we have shown that fetal membranes exhibit dynamic regulation of *Prok1* prior to the up-regulation of other pro-inflammatory mediators before parturition. This suggests an alternate as yet unidentified role similar to that in fetal maturation processes in term parturition in the mouse. We have also demonstrated differences between human and mouse prokineticin expression and regulation in response to LPS in uteroplacental tissues, demonstrating that caution should be applied when using mouse models of PTD. Lastly, we have also contributed to the building body of evidence that PTD is not simply the premature induction of the same pro-inflammatory cascades seen at term by showing the difference in regulation of chemokines at term and in response to LPS, highlighting potentially new targets for the treatment of PTD.

## Supplementary data

This is linked to the online version of the paper at http://dx.doi.org/10.1530/REP-13-0295.

Supplementary Figure

## Figures and Tables

**Table 1:** Treatments and sample size for mouse model of PTL.

**Treatment group**	**Number of dams**
Natural delivery	5
Saline	15
LPS	10
PROK1	16

**Table 2:** Treatments and sample size for gestational tissue and *in vivo* experiments.

**Tissue sample**	**Stage of gestation/treatment**	**Number of dams/samples collected per dam**
Fetal membranes	D16 gestation	10/1
Fetal membranes	D17 gestation	9/1
Fetal membranes	D18 gestation	10/1
Fetal membranes	D19 gestation	10/1
Fetal membranes	D17 6 h saline	4/3
Fetal membranes	D17 6 h LPS	4/3
Fetal membranes	D17 6 h PROK1	6/3
Amniotic fluid	D17 6 h saline	4/4–6
Amniotic fluid	D17 6 h LPS	4/4–6
Amniotic fluid	D17 6 h PROK1	≥5/4–6

**Table 3:** Sequences of mouse primers and probes used in QPCR.

**Gene**	**Primer/probe sequence** (5′–3′)	**Control tissue**
*Prok1*	F: GAAGCCACAAGATCCCCTTCT	D19 fetal membrane
	R: TGCCGTCCGGGAACCT	
	Probe (FAM/TAM): AAACGCCAACACCATACCTGTCCCTG	
*Prokr1*	F: TGGCCCGCTACAAAAAGCT	Adult liver
	R: CCACGAGGAAGTCTGAAATGG	
	Probe (FAM/TAM): CGCAACCTCACCAACCTGCTTATCG	
*Il6*	F: CCACGGCCTTCCCTACTTC	D19 fetal membrane
	R: TGCACAACTCTTTTCTCATTCCA	
	Probe (FAM/TAM): TCACAGAGGATACCACTCCCAACAGACCTG	
*Ptgs2*	F: GCTTCGGGAGCACAACAG	D19 fetal membrane
	R: TGGTTTGGAATAGTTGCTC	
	Probe (FAM/TAM): TGTGCGACATACTCAAGCA	
*Actb*	F: GCTTCTTTGCAGCTCCTTCGT	NA
	R: GCGCAGCGATATCGTCATC	
	Probe (JOE/TAM): CACCCGCCACCAGTTCGCCAT	

**Table 4:** Mouse ABI TaqMan gene expression assays used in QPCR.

**Gene**	**ABI assay code**	**Control tissue**
*Prok2*	Mm01182450_g1	D16 uterus
*Prokr2*	Mm00769571_m1	D16 uterus
*Cxcl2*	Mm00436450_m1	D19 fetal membrane
*Cxcl5*	Mm00436451_g1	D19 fetal membrane
*Il1b*	Mm01336189_m1	D19 uterus
*Tnf*	Mm00443258_m1	D19 uterus
*Ptgs1*	Mm00477214_m1	D19 uterus

**Table 5:** Fold changes in relative expression of pro-inflammatory mediators in mouse fetal membranes between D17 (*n*=9) and D19 (*n*=10) of pregnancy, between 6-h saline (*n*=4) and PROK1 (*n*=6) intrauterine injection, and between 6-h saline and LPS (*n*=4) intrauterine injection.

**Gene**	**Endogenous D17 vs 19**	**Saline vs PROK1**	**Saline vs LPS**
*Ptgs2*	1.73	2.61	13.74
*Il6*	12.04	18.68	62.80
*Il1b*	11.08	17.74	81.43
*Tnf*	2.94	12.54	12.76
*Cxcl2*	90.19	37.52	290.67
*Cxcl5*	440.16	22.45	62.26
