# Supplementary material for: Prokineticin 1 induces a pro-inflammatory response in murine fetal membranes but does not induce preterm delivery
Source: Reproduction. 2013 Aug 13;146(6):581–91. doi: 10.1530/REP-13-0295 (PMC3805954; doi:10.1530/REP-13-0295)
Supplement: Supplementary Figure [file supp_146_6_581__index.html]

Supplementary Figure 

# Prokineticin 1 induces a pro-inflammatory response in murine fetal membranes but does not induce preterm delivery

## Supplementary Figure

**Files in this Data Supplement:**

- Supplementary Figure 1 - *Prok1* mRNA expression is not significantly upregulated preceding labor in murine uterus or placenta. QPCR expression analysis of *Prok1* and *Prokr1* in D16-19 murine uterus (A and B) and placenta (C and D) reveals that *Prok1* is not regulated across D16–19 but *Prokr1* is significantly downregulated on D19. The graphs show individual values for each sample (one membrane analysed per mouse), mean expression levels are in arbitrary units normalised to *ActB* mRNA, error bars represent ± SEM (ANOVA). D16 (n=4), D17 (n=5), D18 (n=5) and D19 (n=5). (PDF 278 KB)
- Supplementary Figure 2 - *Prok2* and *Prokr2* mRNA is expressed in murine uteroplacental tissues preceding labor. QPCR expression analysis of *Prok2* (A, C and E) and Prokr2 (B, D and F) in D16–19 murine uteroplacental tissues reveals the only change in regulation to occur in *Prok2* expression on D18 with a small but significant increase. The graphs show individual values for each sample, mean expression levels are in arbitrary units normalised to *ActB* mRNA, error bars represent ± SEM (ANOVA). D16 (n=4), D17 (n=5), D18 (n=5) and D19 (n=5) except for the fetal membranes where D16 (n=10), D17 (n=9), D18 (n=10) and D19 (n=10). (PDF 289 KB)
- Supplementary Figure 3 - Pro-inflammatory mediator expression increases on D19 of pregnancy in murine fetal membranes. QPCR mRNA expression analysis of *Ptgs2*, *Il6*, *Il1B*, *Tnf*, *Cxcl2*, *Cxcl5* and *Ptgs1* in D16–19 uteroplacental tissues reveals significant upregulation on the day prior to labor (D19). The graphs show individual values for each sample, mean expression levels are in arbitrary units normalised to *ActB* mRNA, error bars represent ± SEM (ANOVA). D16 (n=10), D17 (n=9), D18 (n=10) and D19 (n=10). (PDF 311 KB)
