# Supplementary figures and images for: Prokineticin 1 induces a pro-inflammatory response in murine fetal membranes but does not induce preterm delivery
Source: Reproduction. 2013 Aug 13;146(6):581–91. doi: 10.1530/REP-13-0295 (PMC3805954; doi:10.1530/REP-13-0295)

## Uterus

**A**

*Prok1*

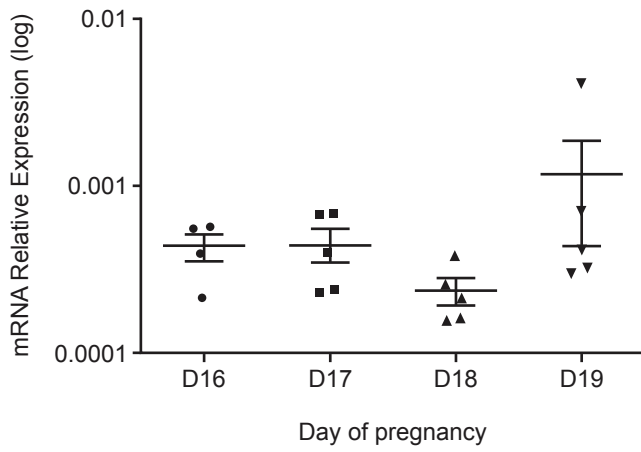

**B**

*Prokr1*

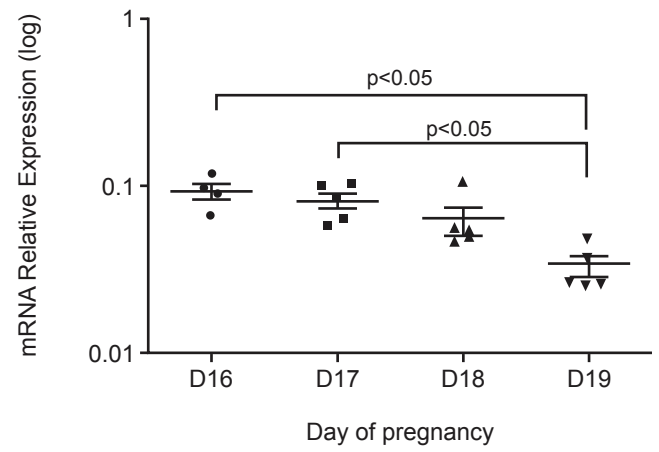

## Placenta

**C**

*Prok1*

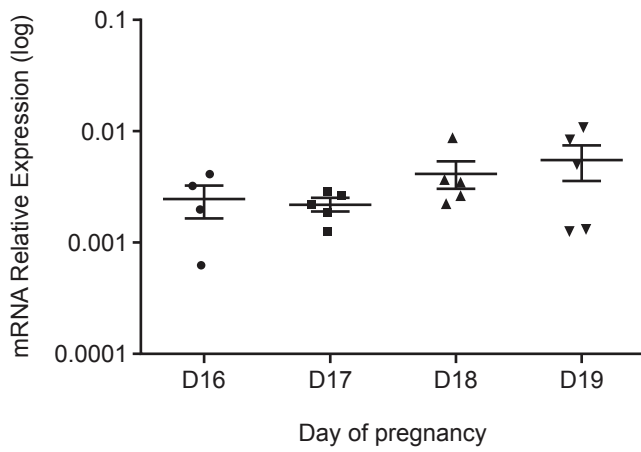

**D**

*Prokr1*

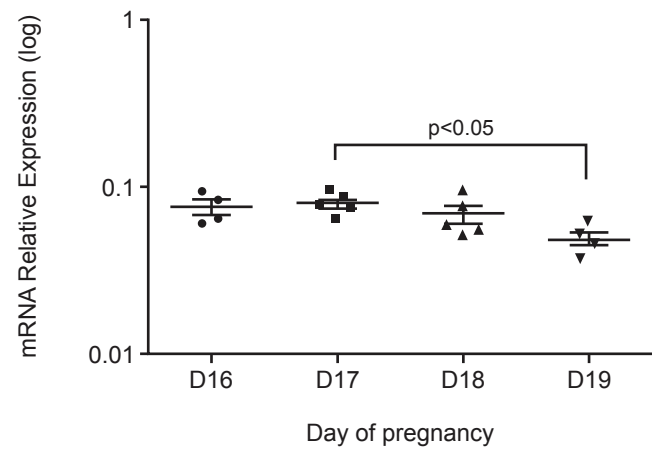

Supplement: Supplementary Figure [file supp_REP-13-0295_Supplementary_figure_1.pdf]

## Fetal membranes

**A**

*Prokr2*

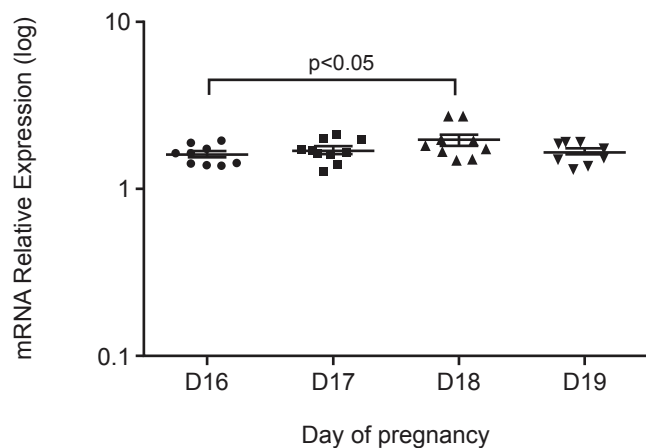

**B**

*Prokr2*

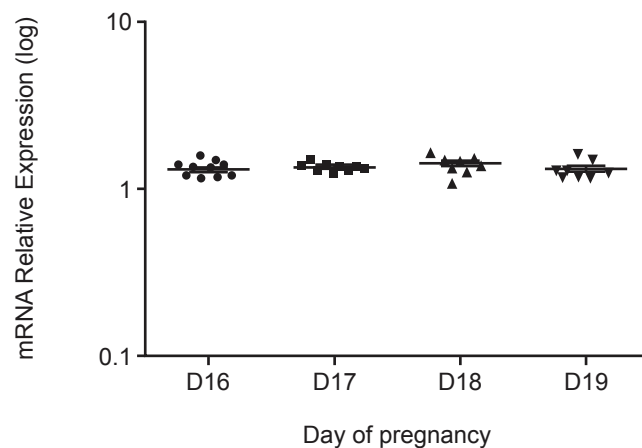

## Uterus

**C**

*Prokr2*

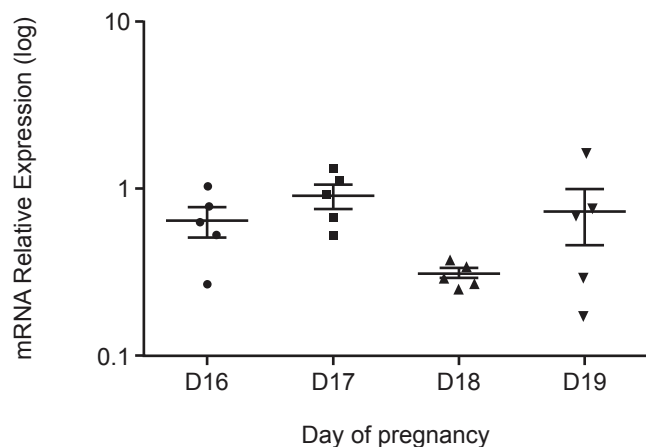

**D**

*Prokr2*

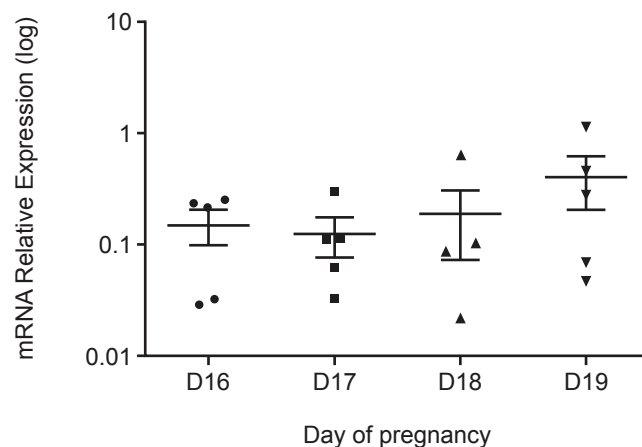

## Placenta

**E**

*Prokr2*

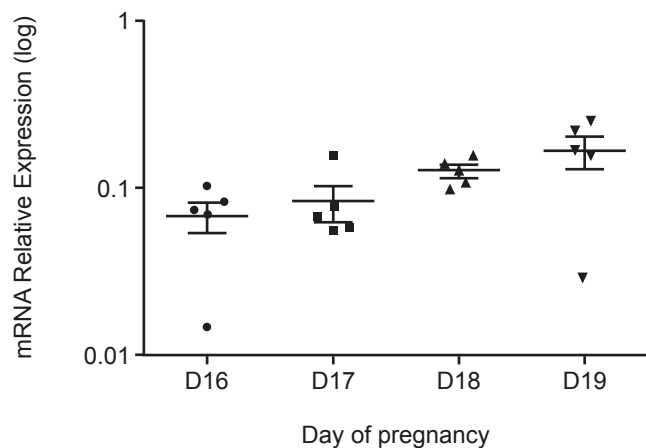

**F**

*Prokr2*

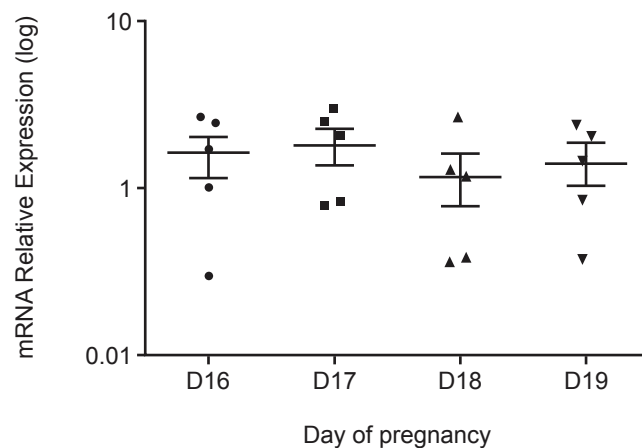

Supplement: Supplementary Figure [file supp_REP-13-0295_Supplementary_figure_2.pdf]

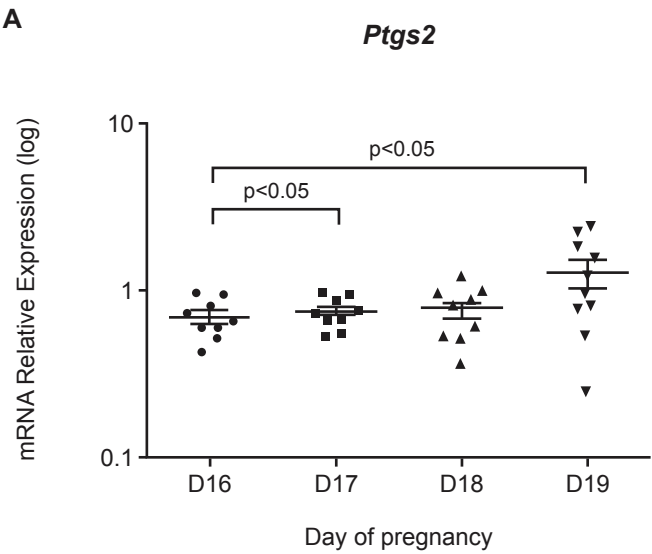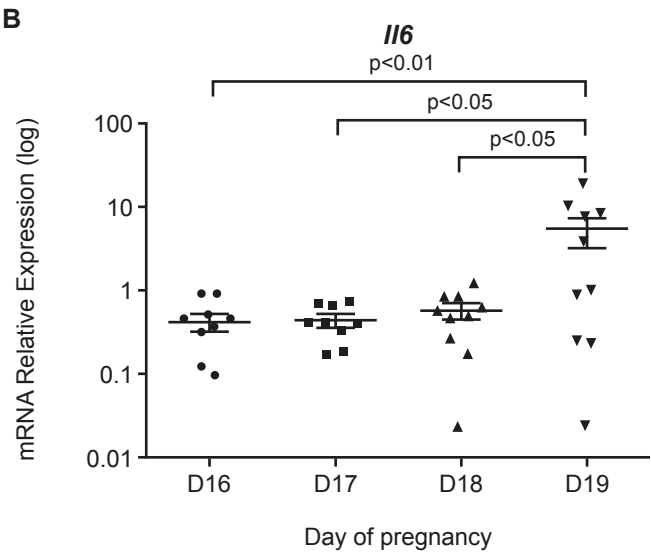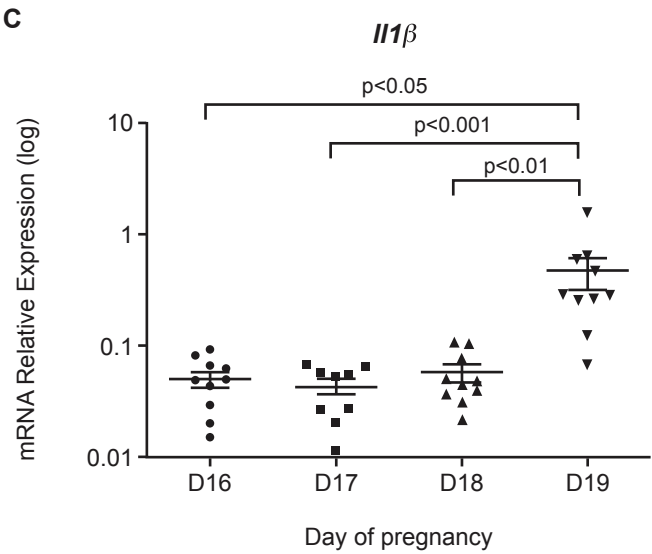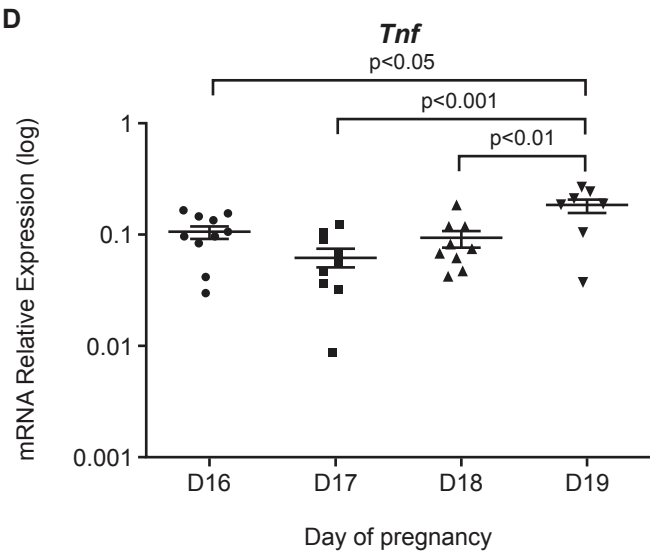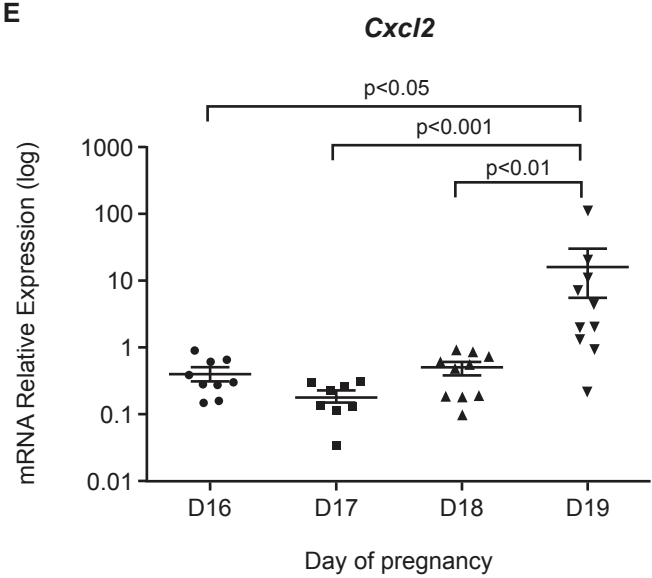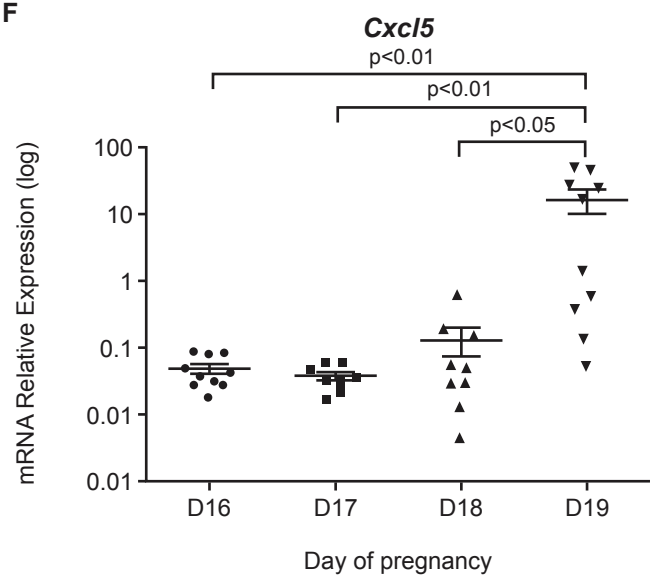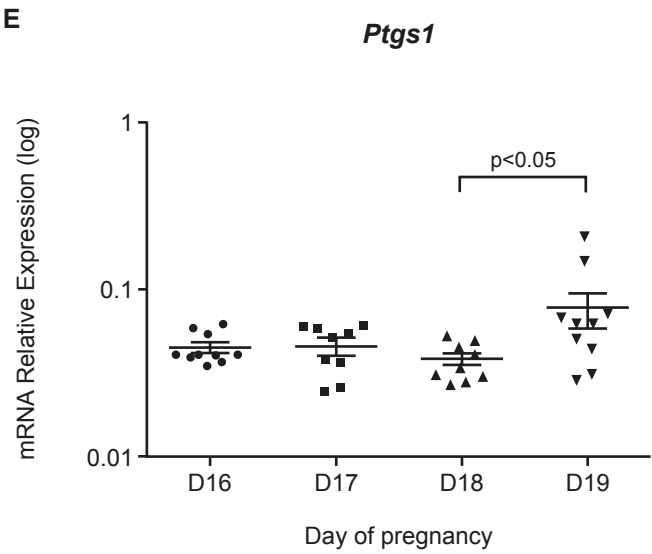

Supplement: Supplementary Figure [file supp_REP-13-0295_Supplementary_figure_3.pdf]
